# Supplementary material for: Parental hesitancy on COVID-19 vaccination of children under the age of 16: A cross-sectional mixed-methods study among factory workers
Source: PLoS One. 2025 Jun 26;20(6):e0327056. doi: 10.1371/journal.pone.0327056 (PMC12200862; doi:10.1371/journal.pone.0327056)
Supplement: S1 File — (PDF) [file pone.0327056.s001.pdf]

Parental hesitancy on COVID-19 vaccination of children under the age of 16: A cross-sectional mixed-methods study among factory workers

Questionnaires

Code: ____/ ____/ ____

Section 1: Socio-economic characteristics

No.	Question	
1	Sex<br>☐ Male (Father)	☐ Female (Mother)
2	Age (completed years) ______________________________	
3	Educational status<br>☐ Illiterate<br>☐ Primary school passed<br>☐ High school passed<br>☐ Postgraduate	☐ Read and write<br>☐ Middle school passed<br>☐ Graduate
4	Current department<br>☐ Administration<br>☐ Production	☐ Management
5	Monthly income (Kyat) ______________________________	
6	Number of household members ______________________________	

Section 2: Status of previous COVID-19 infection and vaccination of the respondents

No.	Question	
1	Have you been infected with the SARS-CoV-2 since the beginning of the pandemic?	
	☐ Yes	☐ No
2	Have your partner's been infected with the SARS-CoV-2 since the beginning of the pandemic?	
	☐ Yes	☐ No
3	Have any of your children been infected with the SARS-CoV-2?	
	☐ Yes	☐ No
4	Did you receive the COVID-19 vaccine?	
	☐ Yes	☐ No
5	Did your partner get the COVID-19 vaccine?	
	☐ Yes	☐ No
6	The main source of information on COVID-19 vaccine is	
	☐ Television	☐ Radio
	☐ Social media	☐ Health care worker
	☐ Friends	☐ Newspaper

Section 3: Attitudes towards COVID-19 vaccination

No.	Statement	SA	A	N	D	SD
1	If I get vaccinated, I will have lesser chance of getting COVID-19 infection.	☐	☐	☐	☐	☐
2	If more people in a society get vaccinated, the number of people getting COVID-19 infection will be reduced.	☐	☐	☐	☐	☐
3	COVID-19 can be controlled if more people get vaccinated.	☐	☐	☐	☐	☐
4	Those who get vaccinated prevent transmission of the disease to other family members.	☐	☐	☐	☐	☐
5	If I do not get vaccinated and catch COVID-19 infection, then disease will be more severe as compared to any vaccinated person.	☐	☐	☐	☐	☐
6	If I don’t get vaccinated and catch COVID-19 infection, I might suffer from other health problems also.	☐	☐	☐	☐	☐
7	COVID-19 infection causes minor illness only.	☐	☐	☐	☐	☐
8	People who exercise and eat healthy foods do not get serious COVID-19 infection.	☐	☐	☐	☐	☐
9	A vaccinated person is fully protected against COVID-19 disease.	☐	☐	☐	☐	☐
10	Vaccination is the safest way to protect oneself from COVID-19 infection.	☐	☐	☐	☐	☐
11	Available COVID-19 vaccine does not contain harmful substances.	☐	☐	☐	☐	☐
12	I do not get vaccinated because I concern about the COVID-19 vaccine’s side effects.	☐	☐	☐	☐	☐
13	Suspicious about the COVID-19 vaccination may depend on the sources of information.	☐	☐	☐	☐	☐
14	I do not need the vaccine if I take all precautions such as wearing of mask and social distancing etc.	☐	☐	☐	☐	☐
15	I do not need the vaccine as I am very healthy.	☐	☐	☐	☐	☐
16	I prefer not to get vaccinated as I needles scare me.	☐	☐	☐	☐	☐

Section 4: The Oxford COVID-19 vaccine hesitancy scale (OC19-VHS)

No.	Question
1	If factory's clinic noticed me to come and receive a COVID-19 vaccine for my child, I would:<br>☐ Make an appointment straight away<br>☐ Make an appointment<br>☐ Wait until a reminder<br>☐ Wait until many reminders<br>☐ Never make an appointment
2	If I had (or do have) a child and the school asked for permission to vaccinate my child for COVID-19, I would:<br>☐ Definitely give permission<br>☐ Probably give permission<br>☐ Possibly give permission<br>☐ Probably not give permission<br>☐ Definitely not give permission
3	If a COVID-19 vaccine was available at my local pharmacy to vaccinate my child, I would:<br>☐ Get it as soon as possible<br>☐ Get it when I have time<br>☐ Delay getting it<br>☐ Avoid getting it for as long as possible<br>☐ Never get it
4	If our children were all required by the government to take a COVID-19 vaccine, I would:<br>☐ Definitely take it<br>☐ Accept it<br>☐ Be hesitant and delay it<br>☐ Refuse<br>☐ Strongly refuse

5	I would describe myself as:<br>☐ Eager to get a COVID-19 vaccine<br>☐ Willing to get the COVID-19 vaccine<br>☐ Not bothered about getting the COVID-19 vaccine<br>☐ Unwilling to get the COVID-19 vaccine<br>☐ Anti-vaccination for COVID-19
6	Taking a COVID-19 vaccination is:<br>☐ Really important<br>☐ Important<br>☐ Neither important nor unimportant<br>☐ Unimportant<br>☐ Really unimportant
7	With regards to a COVID-19 vaccine if I had (or do have) a child:<br>☐ I would definitely get them vaccinated<br>☐ I would probably get them vaccinated<br>☐ I may or may not get them vaccinated<br>☐ I would not get them vaccinated<br>☐ I would make sure that they were not vaccinated
