# Supplementary material for: Parental hesitancy on COVID-19 vaccination of children under the age of 16: A cross-sectional mixed-methods study among factory workers
Source: PLoS One. 2025 Jun 26;20(6):e0327056. doi: 10.1371/journal.pone.0327056 (PMC12200862; doi:10.1371/journal.pone.0327056)
Supplement: S2 File — (PDF) [file pone.0327056.s002.pdf]

အသက်(၁၆)နှစ်အောက်ကလေးရှိသော မိဘများ၏သားသမီးများအား ကိုဗစ်-၁၉ရောဂါ ကာကွယ်ဆေးထိုးရန် နှောင့်နှေးခြင်း (သို့) ငြင်းဆန်ခြင်း အခြေအနေကို မိဘများတွင် လေ့လာစမ်းစစ်ခြင်း

မေးခွန်းလွှာ

Code: ____/ ____/ ____

အပိုင်း ၁။ ဖြေဆိုသူ၏ ကိုယ်ရေးအချက်အလက်များနှင့် လူမှုစီးပွားရေးဆိုင်ရာ အချက်အလက်များ

စဉ်	မေးခွန်း
၁	လိင်<br>☐ ကျား (အဖေ) ☐ မ (အမေ)
၂	အသက် (ပြည့်ပြီးအသက်) ____
၃	ပညာအရည်အချင်း<br>☐ စာမတတ်ပါ ☐ ရေး/ဖတ်<br>☐ မူလတန်းအောင် ☐ အလယ်တန်းအောင်<br>☐ အထက်တန်းအောင် ☐ ဘွဲ့ရ<br>☐ ဘွဲ့လွန်
၄	လက်ရှိအလုပ်အကိုင်နေရာ/ဌာန<br>☐ အုပ်ချုပ်မှုဌာန ☐ စီမံဌာန<br>☐ ထုတ်လုပ်ရေးဌာန
၅	မိသားစု၏ တစ်လဝင်ငွေ (ကျပ်) ____
၆	မိသားစု အရေအတွက် ____

အပိုင်း ၂။ ကိုဗစ်-၁၉ ရောဂါကူးစက်ခံရမှု့အခြေအနေနှင့် ကာကွယ်ဆေးထိုးနှံခြင်းအခြေအနေများ

စဉ်	မေးခွန်း	
၁	မိမိကိုယ်တိုင် ကိုဗစ်-၁၉ ရောဂါ ကူးစက်ခံရခြင်း ရှိပါသလား။	
	☐ ကူးစက်ခံရပါသည်။	☐ ကူးစက်ခြင်း မခံရပါ။
၂	ဇနီး/ခင်ပွန်း ဖြစ်သူသည်လည်း ကိုဗစ်-၁၉ ရောဂါ ကူးစက်ခံရခြင်း ရှိပါသလား။	
	☐ ကူးစက်ခံရပါသည်။	☐ ကူးစက်ခြင်း မခံရပါ။
၃	ဖြေဆိုသူ၏ သား/သမီးများသည်လည်း ကိုဗစ်-၁၉ ရောဂါ ကူးစက်ခံရခြင်း ရှိပါသလား။	
	☐ ကူးစက်ခံရပါသည်။	☐ ကူးစက်ခြင်း မခံရပါ။
၄	မိမိကိုယ်တိုင် ကိုဗစ်-၁၉ ရောဂါ ကာကွယ်ဆေး ထိုးနှံပြီးစီးမှု့ရှိပါသလား။	
	☐ ထိုးနှံပြီးပါပြီ။	☐ ထိုးနှံခြင်းမရှိပါ။
၅	ဇနီး/ခင်ပွန်းဖြစ်သူသည် ကိုဗစ်-၁၉ ရောဂါ ကာကွယ်ဆေး ထိုးနှံပြီးစီးမှု့ရှိပါသလား။	
	☐ ထိုးနှံပြီးပါပြီ။	☐ ထိုးနှံခြင်းမရှိပါ။
၆	ကိုဗစ်-၁၉ ရောဂါကာကွယ်ဆေးနှင့်ပတ်သက်သည့်သတင်းအချက် အလက်များကို မည်သည့်နေရာမှလေ့လာသိရှိပါသနည်း။	
	☐ ရုပ်မြင်သံကြား	☐ ရေဒီယို
	☐ လူမှုကွန်ယက်	☐ ကျန်းမာရေးဝန်ထမ်းများ
	☐ သူငယ်ချင်း/မိတ်ဆွေများ	☐ သတင်းစာများ

အပိုင်း ၃။ ကိုရိုနာဗိုင်းရပ်(စ်)-၁၉ရောဂါကာကွယ်ဆေးထိုးနှံခြင်းနှင့် ပတ်သက်သည့်ခံယူချက်များ

စဉ်	မေးခွန်း	အလွန်သဘောတူပါသည်။	သဘောတူပါသည်။	မသိပါ။	သဘောမတူပါ။	အလွန်သဘောမတူပါ။
၁	ကျွန်ုပ်သည် ကာကွယ်ဆေးထိုးထားပါက ကိုဗစ်-၁၉ရောဂါ ကူးစက်ခံရရန် အခွင့်အလမ်းနည်းပါသည်။	☐	☐	☐	☐	☐
၂	လူထုအတွင်းကာကွယ်ဆေးထိုးနှံသူအရေအတွက်များ လာလျှင်ကိုဗစ်-၁၉ ရောဂါကူးစက်ခံရသူ အရေအတွက် လျော့ကျသွားမည်ဖြစ်ပါသည်။	☐	☐	☐	☐	☐
၃	ကာကွယ်ဆေးထိုးနှံမှုအရေအတွက်များလာသည်နှင့် အမျှ ကိုဗစ်-၁၉ရောဂါ ကို ထိန်းချုပ်နိုင်မည် ဖြစ်ပါသည်။	☐	☐	☐	☐	☐
၄	ကျွန်ုပ်သည်ကိုဗစ်-၁၉ ရောဂါကာကွယ် ဆေးထိုးထားပါက မိမိ၏ မိသားစုဝင်များထံ ရောဂါကူးစက်ခံရမှုကိုကာကွယ်နိုင်ပါသည်။	☐	☐	☐	☐	☐
၅	ကာကွယ်ဆေးထိုးနှံမှုမရှိပဲကိုဗစ်-၁၉ ရောဂါ ကူးစက်ခံ ရလျှင် ကာကွယ်ဆေး ထိုးထားသူများနှင့်နှိုင်းယှဉ်ပါက ရောဂါပိုမို ပြင်းထန်နိုင်ပါသည်။	☐	☐	☐	☐	☐
၆	ကာကွယ်ဆေးထိုးနှံမှုမရှိပဲကိုဗစ်၁၉ရောဂါကူးစက်ခံရ လျှင် အခြားကျန်းမာရေးပြဿနာများပါ ထပ်မံတွေ့ကြုံ ခံစားနိုင်ပါသည်။	☐	☐	☐	☐	☐
၇	ကိုဗစ်-၁၉ ရောဂါကူးစက်ခံရမှုသည် သာမန် ဖျားနာခြင်းကိုသာ ဖြစ်ပေါ်စေသည်။	☐	☐	☐	☐	☐
၈	လေ့ကျင့်ခန်းပုံမှန်ပြုလုပ်၍ကျန်းမာရေးနှင့်ညီညွတ် သောအစားအစာများကိုပုံမှန်စားသုံးသူများတွင် ကိုဗစ်-၁၉ ရောဂါ ကူးစက်ခံရပါက ရောဂါပြင်းထန်မှု မရှိပါ။	☐	☐	☐	☐	☐

၉	ကာကွယ်ဆေးထိုးထားသူများသည်ကိုဗစ်-၁၉ရောဂါ ကူးစက်ခံရမှုကို အပြည့်အဝကာကွယ်နိုင်ပါသည်။	☐	☐	☐	☐	☐
၁၀	ကာကွယ်ဆေးထိုးနှံခြင်းသည်မိမိအား ကိုဗစ်၁၉ရောဂါ ကူးစက်ခံရမှုမှကာကွယ်နိုင်ရန် အလုံခြုံဆုံးနည်းလမ်း ဖြစ်သည်။	☐	☐	☐	☐	☐
၁၁	လက်ရှိထိုးနှံပေးနေသော ကိုဗစ်-၁၉ ရောဂါ ကာကွယ်ဆေး များတွင်အန္တရာယ်ရှိသော အရာများမပါဝင်ပါ။	☐	☐	☐	☐	☐
၁၂	ကျွန်ုပ်သည်ကိုဗစ်-၁၉ရောဂါကာကွယ်ဆေး၏ ဘေးထွက် ဆိုးကျိုးများအပေါ် စိုးရိမ်ပူပန်မှု ရှိသောကြောင့်ကာကွယ်ဆေးမထိုးလိုပါ။	☐	☐	☐	☐	☐
၁၃	ကိုဗစ်-၁၉ ရောဂါကာကွယ်ဆေးထိုးနှံမှုအား သံသယဖြစ်ခြင်းသည် သတင်းအချက်အလက်များ ရရှိမှုအပေါ် မူတည်ပါသည်။	☐	☐	☐	☐	☐
၁၄	ကြိုတင်ကာကွယ်မှုများ (ဥပမာ-နှာခေါင်းစည်း တပ်ဆင်ခြင်းနှင့် လူစုလူဝေးများအားရှောင်ရှားခြင်း) ပြုလုပ်ခြင်းဖြင့် ကိုဗစ်-၁၉ ရောဂါကာကွယ်ဆေး ထိုးနှံရန်မလိုအပ်ပါ။	☐	☐	☐	☐	☐
၁၅	ကျွန်ုပ်သည် ကျန်းမာရေးကောင်းမွန်သောကြောင့် ကိုဗစ်-၁၉ရောဂါ ကာကွယ်ဆေး ထိုးနှံရန် မလိုအပ်ပါ။	☐	☐	☐	☐	☐
၁၆	ကျွန်ုပ်သည် ဆေးထိုးအပ်ကြောက်ခြင်းကြောင့် ကိုဗစ်-၁၉ရောဂါ ကာကွယ်ဆေး မထိုးနှံလိုပါ။	☐	☐	☐	☐	☐

အပိုင်း ၄။ မိဘများ၏ သားသမီးများအား ကိုဗစ်-၁၉ ရောဂါကာကွယ်ဆေးထိုးခြင်းအပေါ် စိုးရိမ်ပူပန်မှု

စဉ်	မေးခွန်း
၁	အကယ်၍စက်ရုံရှိဆေးခန်းမှမိမိ၏သား/သမီးများအားကိုဗစ်-၁၉ ရောဂါ ကာကွယ်ဆေးထိုးနှံရန်အကြောင်းကြားပါက ကျွန်တော်/ကျွန်မသည်-<br>☐ ချက်ချင်းစာရင်းပေးပါမည်။<br>☐ စာရင်းပေးပါမည်။<br>☐ နောက်တကြိမ်ထပ်မံအကြောင်းကြားခြင်းကိုစောင့်ဆိုင်းပါမည်။<br>☐ အကြိမ်ရေများစွာ အကြောင်းကြားခြင်းကို စောင့်ဆိုင်းပါမည်။<br>☐ စာရင်းလုံးဝမပေးပါ။
၂	မိမိ၏သား/သမီးများတက်ရောက်နေသည့်ကျောင်းမှကိုဗစ်-၁၉ ရောဂါ ကာကွယ်ဆေးထိုးနှံရန်ခွင့်ပြုချက်တောင်းခံလာပါက ကျွန်တော်/ကျွန်မသည်-<br>☐ လုံးဝခွင့်ပြုပါမည်။<br>☐ ခွင့်ပြုပါမည်။<br>☐ ခွင့်ပြုချက်ပေးနိုင်ပါသည်။<br>☐ ခွင့်မပြုပါ၊<br>☐ လုံးဝခွင့်မပြုပါ။
၃	ပုဂ္ဂလိကဆေးခန်း/ဆေးရုံများတွင်မိမိ၏သား/သမီးများအား ကိုဗစ်-၁၉ရောဂါ ကာကွယ်ဆေးထိုးနှံနိုင်ပါက ကျွန်တော်/ကျွန်မသည်-<br>☐ ချက်ချင်းသွားရောက်ထိုးနှံပါမည်။<br>☐ မိမိအားလပ်သောအချိန် မှသာသွားရောက်ထိုးနှံပါမည်။<br>☐ အချိန်ရမှသာသွားရောက်ထိုးနှံပါမည်။<br>☐ မထိုးနှံသေးပဲ တတ်နိုင်သမျှနေပါမည်။<br>☐ မည်သည့်အခါမှမထိုးပါ။
၄	မိမိ၏သား/သမီးများအား အစိုးရမှ ကိုဗစ်-၁၉ရောဂါ ကာကွယ်ဆေး မဖြစ်မနေထိုးနှံရန် ညွှန်ကြားပါက ကျွန်တော်/ကျွန်မသည်-<br>☐ လုံးဝထိုးနှံပါမည်။<br>☐ ထိုးနှံပါမည်။<br>☐ မထိုးနှံသေးပဲ တတ်နိုင်သမျှနေပါမည်။<br>☐ ငြင်းဆိုပါမည်။<br>☐ အခိုင်အမာ ငြင်းဆိုပါမည်။

၅	ကိုဗစ်-၁၉ရောဂါ ကာကွယ်ဆေးထိုးနှံခြင်းအပေါ် မိမိ၏ဆန္ဒမှာ-<br>☐ ထိုးနှံရန်အလွန်ဆန္ဒရှိသူဖြစ်ပါသည်။<br>☐ ထိုးနှံရန် ဆန္ဒရှိသူဖြစ်ပါသည်။<br>☐ ထိုးနှံရန်မည်သည့်သဘောထားမျှမရှိသူဖြစ်ပါသည်။<br>☐ ထိုးနှံရန်ဆန္ဒမရှိသူဖြစ်ပါသည်။<br>☐ ထိုးနှံရန်လုံးဝဆန္ဒမရှိသူဖြစ်ပါသည်။
၆	ကိုဗစ်-၁၉ရောဂါ ကာကွယ်ဆေးထိုးနှံခြင်းသည်-<br>☐ အလွန်အရေးကြီးပါသည်။<br>☐ အရေးကြီးပါသည်။<br>☐ မည်သည့်ထင်မြင်ယူဆချက်မှမရှိပါ။<br>☐ အရေးမကြီးပါ။<br>☐ လုံးဝအရေးမကြီးပါ။
၇	မိမိ၏သား/သမီးများအားကိုဗစ်-၁၉ရောဂါကာကွယ်ဆေးထိုးနှံမှုအပေါ်မိမိ၏စိတ်ဆန္ဒသဘောထားမှာ-<br>☐ လုံးဝထိုးနှံပေးလိုပါသည်။<br>☐ ထိုးနှံပေးလိုပါသည်။<br>☐ မည်သည့်သဘောထားမျှမရှိပါ။<br>☐ ထိုးနှံမပေးပါ။<br>☐ လုံးဝထိုးနှံမပေးပါ။
