# Supplementary material for: Parental hesitancy on COVID-19 vaccination of children under the age of 16: A cross-sectional mixed-methods study among factory workers
Source: PLoS One. 2025 Jun 26;20(6):e0327056. doi: 10.1371/journal.pone.0327056 (PMC12200862; doi:10.1371/journal.pone.0327056)
Supplement: S3 File — (PDF) [file pone.0327056.s003.pdf]

In-depth interview guide

Interviewee category: Non-hesitant group

Code: C19VH ☐ ☐

Sex ________________

Age ________________ years

===

Why are you willing to vaccinate your children against COVID-19? Can you explain more?

Probes:

- Due to improvement of body immunity?
- Due to decreasing the cases detection and death rate?
- Due to free of charges?
- Due to safety and effectiveness?
- Due to prevention from being infected with COVID-19?

Interviewee category: Hesitant group

Code: C19VH ☐☐

Sex ________________

Age ________________ years

==

Why are you unwilling to vaccinate your children against COVID-19? Can you explain more?

Probes:

- Due to concern the side effects of the COVID-19 vaccines for children?
- Due to unsure vaccine safety?
- Due to unsure vaccine effectiveness?
- Due to not trust the origin of the vaccine (company or country)?
- Due to the child’s underlying diseases?
- Due to concern the tolerance of side effect by age of the child?
- Due to anti-vaccination infodemic on social media?
- Due to religious belief?
