# Supplementary material for: Parental hesitancy on COVID-19 vaccination of children under the age of 16: A cross-sectional mixed-methods study among factory workers
Source: PLoS One. 2025 Jun 26;20(6):e0327056. doi: 10.1371/journal.pone.0327056 (PMC12200862; doi:10.1371/journal.pone.0327056)
Supplement: Attachment — Submitted filename: Response to Reviewers.pdf [file pone.0327056.s006.pdf]

Responses to editor and reviewers

PONE-D-25-10068R1

Parental hesitancy on COVID-19 vaccination of children under the age of 16: A cross-sectional mixed-methods study among factory workers

PLOS ONE

Revised manuscript on April 14, 2025

Dear Prof. Harapan Harapan,

I would like to thank you for the opportunity to revise and resubmit our manuscript titled "Parental hesitancy on COVID-19 vaccination of children under the age of 16: A cross-sectional mixed-methods study among factory workers". We are grateful to you and the reviewers for their helpful comments on our manuscript. It is our belief that the manuscript is substantially improved after making the suggested edits.

Followings this letter are the editor and reviewers' comments with our responses in point by point, including how and where the text was modified. Changes made in the revised manuscript are marked using track changes. The revision has been developed in consultation with all authors, and each author has given approval to the final form of this revision.

Thank you again for consideration of our revised manuscript.

Sincerely,

Ye Minn Htun

Health and Disease Control Unit

Nay Pyi Taw 15011, Myanmar

Journal Requirements

When submitting your revision, we need you to address these additional requirements.

Comment 1: Please ensure that your manuscript meets PLOS ONE's style requirements, including those for file naming. The PLOS ONE style templates can be found at

https://journals.plos.org/plosone/s/file?id=wjVg/PLOSOne_formatting_sample_main_body.pdf and

https://journals.plos.org/plosone/s/file?id=ba62/PLOSOne_formatting_sample_title_authors_affiliations.pdf

Response: Thanks for this comment and we made sure that our manuscript meets PLOS ONE’s style requirements.

Comment 2: Please provide additional details regarding participant consent. In the ethics statement in the Methods and online submission information, please ensure that you have specified what type you obtained (for instance, written or verbal, and if verbal, how it was documented and witnessed). If your study included minors, state whether you obtained consent from parents or guardians. If the need for consent was waived by the ethics committee, please include this information.

Once you have amended this/these statement(s) in the Methods section of the manuscript, please add the same text to the “Ethics Statement” field of the submission form (via “Edit Submission”).

For additional information about PLOS ONE ethical requirements for human subjects research, please refer to http://journals.plos.org/plosone/s/submission-guidelines#loc-human-subjects-research.

Response: Thank you for this comment and the manuscripts conformed the STROBE guideline for the observational studies. We revised in manuscript and "Ethics Statement" field of the submission form as the following:

"Firstly, the participants were explained about the nature of the study and then written informed consent was taken from the participants before data collection." *(Materials and methods, Page No. 13, Line No. 287-288)*

Comment 3: We note that there is identifying data in Table 5. Prior to sharing human research participant data, authors should consult with an ethics committee to ensure data are shared in accordance with participant consent and all applicable local laws.

Data sharing should never compromise participant privacy. It is therefore not appropriate to publicly share personally identifiable data on human research participants. The following are examples of data that should not be shared:

-Name, initials, physical address

-Ages more specific than whole numbers

-Internet protocol (IP) address

-Specific dates (birth dates, death dates, examination dates, etc.)

-Contact information such as phone number or email address

-Location data

-ID numbers that seem specific (long numbers, include initials, titled "Hospital ID") rather than random (small numbers in numerical order)

Data that are not directly identifying may also be inappropriate to share, as in combination they can become identifying. For example, data collected from a small group of participants, vulnerable populations, or private groups should not be shared if they involve indirect identifiers (such as sex, ethnicity, location, etc.) that may risk the identification of study participants.

Additional guidance on preparing raw data for publication can be found in our Data Policy (https://journals.plos.org/plosone/s/data-availability#loc-human-research-participant-data-and-other-sensitive-data) and in the following article: http://www.bmj.com/content/340/bmj.c181.long.

Please remove or anonymize all personal information, ensure that the data shared are in accordance with participant consent, and re-upload a fully anonymized data set. Please note that spreadsheet columns with personal information must be removed and not hidden as all hidden columns will appear in the published file.

Response: Thanks for your suggestion. We revised and re-upload the Excel data set file (S4 file) after removing all personal information. Moreover, concerning for the direct or indirect identifiers of data, we removed Table 5 and revised as the following:

"The individual IDIs were performed to explore the reasons for hesitancy of parents on COVID-19 vaccination to their children from the hesitant group (n=6) and the reasons for the non-hesitancy of parents on COVID-19 vaccination to their children from the non-hesitant group (n=6)." *(Results, Page No. 19, Line No. 348-351)*

Review Comments to the Author

Reviewer #2:

A review report of the manuscript entitled “Parental hesitancy on COVID-19 vaccination of children under the age of 16: A cross- sectional mixed-methods study among factory workers”

Comment 1: (page 3, lines 43-44) The opening sentence would be better to rewrite this way: “Thanks to the development of COVID-19 vaccines, now they can be safely and effectively used to guard COVID-19 patients against severe illness, hospitalization, and even mortality.”

Response: Thanks for your suggestion and we rewrite the sentence. *(Abstract, Page No. 3, Line No. 43-45)*

Comment 2: (page 3, line 53) "A total of 170 factory workers having children under the age of 16 in this study" is incomplete. You could say, "A total of 170 factory workers with children under the age of 16 participated in this study."

Response: We would like to thank for this comment. And we revised as follow:

“A total of 170 factory workers with children under the age of 16 participated in this study.” *(Abstract, Page No. 3, Line No. 54)*

Comment 3: (page 5, lines 86-88) This sentence “COVID-19 is continuing to spread around the world with more than 758 million confirmed cases and more than 6.8 million deaths...” also shares the same idea with a similar study by Duta et al. entitled “Essential oil for COVID-19 management: A systematic review of randomized controlled trials” Kindly include this reference to make the sentence stronger.

Response: Thanks for your suggestion. We agreed with it and revised as following:

“COVID-19 is continuing to spread around the world with more than 758 million confirmed cases and more than 6.8 million deaths, at the end of February 2023 [2, 3].” *(Introduction, Page No. 5, Line No. 87-89)*

Comment 4: (page 5, line 105) As authors have defined the acronym of WHO earlier in this manuscript (line 83), there’s no need to give its full definition anymore on the next mention. So, using only WHO here is already enough.

Response: Thanks for this positive comment. We edited it as follow:

“WHO recommended that the countries should continue to accomplish …” *(Introduction, Page No. 5, Line No. 106-107)*

Comment 5: (page 6, lines 112-121) This paragraph “Vaccine hesitancy, which expresses the unwillingness to get vaccines when vaccination services are available and accessible, is one of the greatest threats to global health…” will be more significant if it’s also supported by another relevant study. For example, from Hassan et al. entitled “Global acceptance and hesitancy of COVID-19 vaccination: A narrative review”

Response: Thank you for this suggestion and we added this relevant study for the more significant information.

“Vaccine hesitancy, which expresses the unwillingness to get vaccines when vaccination services are available and accessible, is … suffering and deaths [14, 15].” *(Introduction, Page No. 6, Line No. 112-121)*

Comment 6: (page 8, lines 159-160) The sentence "All parents having children under 16-year-old were working at…" is grammatically incorrect. I would suggest writing it this way "All

parents of children under 16 years old working at Tri Star Tyre Factory were recruited for this study."

Response: We would like to thank for it and revised as follow:

"All parents of children under 16-year-old working at Tri Star tyre factory were recruited for this study." *(Materials and Methods, Page No. 8, Line No. 172-173)*

Comment 7: (page 8, lines 166-168) The authors stated that "In the first section, there were 6 items related socioeconomic characteristics of the parents such as sex, age, level of education, occupational status, monthly family income, and family members." My concern is what kind of data has been taken from "family members". Was that the number of them in a family? Please clarify.

Response: Thanks for the comment to clarify. We mean that this variable is the total number of individuals who are residing in the same housing unit. Therefore, to get more clarify, we edited as follow:

"In the first section, there were 6 items related socioeconomic characteristics of the parents such as sex, age, level of education, occupational status, monthly family income, and total number of household members." *(Materials and Methods, Page No. 8, Line No. 179-182)*

AND

Table 4. *(Results, Page No. 17-18, Line No. 344)*

Comment 8: Authors are suggested to proofread the manuscript after addressing all comments to avoid any typological, grammatical, and lingual mistakes and errors. For example, "questionaries" on page 9 line 182; full stop missing on page 10 line 216; "their children received the had higher attitude scores" on page 27 line 526.

Response: Thanks for pointing them out. We re-checked throughout the manuscript and revised according to your comments as followings:

"The items of the questionnaires for this study were prepared …" *(Materials and Methods, Page No. 9, Line No. 196)*

"…, and "parental COVID-19 vaccine hesitancy-no" (combined unsure and non-hesitant groups)." *(Materials and Methods, Page No. 11, Line No. 233-234)*

"..., parents allowed COVID-19 vaccine to their children had higher attitude scores than those whose children did not receive the vaccine." *(Discussion, Page No. 27, Line No. 548-550)*

Comment 9: (page 11, line 231) Please give the meaning of IDIs abbreviation on the first mention.

Response: Thanks for this comment and we revised as following:

"In the qualitative approach, individual in-depth-interviews (IDIs) guides were explicitly adapted …" *(Materials and Methods, Page No. 11, Line No. 253)*

Comment 10: (page 13, lines 279-280) "In the educational status, (64.1%) participants graduated…" Do the authors mean "bachelor graduate" here?

Response: Thank you for the comment. Graduate in the educational status means the participant who has completed bachelor degree. To more clarify, we added the operational definition of educational status as following:

"The educational status referred to the highest level of education that a participant has successfully completed and it was categorized as primary school level, middle school level, high school level, graduate (completed bachelor degree), and postgraduate (completed advanced degrees such as master or doctoral study)." *(Materials and Methods, Page No. 9-10, Line No. 205-208)*

Comment 11: (page 13, lines 284-285) "…monthly family income of the participants was 317,500 (150,000) kyats with a range of 150,000 to 900,000 kyats…" It would be clearer if authors gave the US dollar estimation of the salary received by the factory workers here. So please add the amount of the money they receive as salary in both kyats and USD.

Response: Thanks for this suggestion. We agreed with this suggestion and described both kyats and USD as following:

"The median (IQR) monthly family income of the participants was 317,500 (150,000) kyats [151.19 (71.43) USD] with a range of 150,000 kyats [71.43 USD] to 900,000 kyats [428.57 USD] and 120 (70.6%) participants had 300,000 kyats [142.86 USD] and above monthly family income." *(Results, Page No. 14, Line No. 303-306)*

And remarked in Table 1 as:

"According to the reference exchange rate of the Central Bank of Myanmar, 1 USD to Myanmar Kyats is 2,100 during data collection period (https://forex.cbm.gov.mm)."

Comment 12: (page 23, lines 430-431) "It showed that acceptance of COVID-19 vaccine hesitancy was increasing in adults and vaccination rates tend to be increasing." This sentence contradicts the previous sentences as the authors used the "hesitancy" word in it. It should be revised.

Response: Thanks for your pointing out and we edited as following:

"It showed that hesitancy to COVID-19 vaccination was decreasing in adults and vaccination rates tend to be increasing." *(Discussion, Page No. 23, Line No. 453-454)*

Comment 13: (page 23, lines 435-436) "In this study, the most trusted source of information for the respondents regarding COVID-19 vaccination was social media (70.0%), followed by

television (55.3%).” This is totally new information, as authors did not mention/report it previously in the methods and results sections.

Response: Thanks for the comment and we have mentioned “the main sources of information” in results section as followings:

“… vaccination status of partners, and main source of information on COVID-19 vaccine.” *(Materials and Methods, Page No. 9, Line No. 185-186)*

“Fig 2 shows the main source of information on the COVID-19 vaccine among the participants: 119 (70.0%) of the respondents got information from social media, 94 (55.3%) from television, …” *(Results, Page No. 15, Line No. 316-319)*

Reviewer #3:

Thank you for the opportunity to review your manuscript entitled “Parental Hesitancy on COVID-19 Vaccination of Children Under the Age of 16: A Cross-Sectional Mixed-Methods Study Among Factory Workers.” I very much enjoyed reading your manuscript.

Summary: This study investigates parental hesitancy toward COVID-19 vaccination for children under 16 among factory workers in Myanmar, utilizing a cross-sectional mixed-methods design. Quantitative data were gathered through structured interviews with 170 factory workers and analyzed using binary logistic regression to determine factors associated with vaccine hesitancy. The qualitative component involved in-depth interviews with 12 participants to explore reasons for hesitancy and non-hesitancy. The results indicate that 18.2% of parents were hesitant, 25.9% were unsure, and 55.9% were non-hesitant. Key factors associated with hesitancy included male gender (AOR: 3.04, 95% CI: 1.35–6.84) and lack of prior SARS-CoV-2 infection (AOR: 2.66, 95% CI: 1.06–6.70). Common concerns included child age, vaccine safety, uncertainty about effectiveness, and distrust in vaccine origin. The

study suggests that health education programs and media campaigns should be strengthened to address misinformation and improve vaccination rates. While the study provides valuable insights into vaccine hesitancy within a specific workforce, further multi-center studies with a larger sample size and comparative analysis across different occupational groups could enhance its generalizability.

Major Comments:

1. Literature Review:

Comment 1: You may expand the discussion on global vaccine hesitancy trends, particularly in occupational settings, to contextualize the study findings.

Response: Thanks for pointing this out and we revised as following:

"Factory workers are essential for driving the production growth and play a crucial role in generating social wealth [16]. The effective COVID-19 vaccination program with the strategies of sustainable vaccination process among factory workers is vital for the recovery of economic sector [17]. Regarding the occupational status, frontline workers such as healthcare workers and workers in customer-facing industries were at higher risk of infection, and therefore these occupations should receive high priority for vaccination [18, 19]. However, the vaccination coverage among industrial workers was usually lower than that of the general population [16]. *(Introduction, Page No. 6, Line No. 122-129)*

Comment 2: You may provide more comparative insights into vaccine hesitancy among factory workers versus the general population in Myanmar or similar settings.

Response: Thanks for this comment. We agreed with it and added as following:

"Based on the findings of a previous study, antivaccine attitude, vaccine origin, education, marital status, having children, participants with chronic diseases were determinants of

COVID-19 vaccine hesitancy among industrial workers [20].” *(Introduction, Page No. 6, Line No. 129-131)*

Comment 3: You may include recent studies on COVID-19 vaccine acceptance among parents to strengthen the theoretical background.

Response: We would like to thank you for this comment. The recent studies on COVID-19 vaccine hesitancy and acceptance among parents were added in the manuscript. We revised to get more clearer as following:

“As per prior studies, the parental vaccine hesitancy to COVID-19 is ranging from 15.1% to 61.9% around the world. Understanding the differences in parental COVID-19 vaccine hesitancy to their children across varying communities and sociodemographic groups plays a critical role in diminishing the burden of the pandemic through administering vaccinations as a preventive behaviour [22-28].” *(Introduction, Page No. 6-7, Line No. 136-140)*

2. Theoretical Contribution:

Comment 4: You may clearly define how this study adds to the existing body of knowledge on vaccine hesitancy in occupational settings.

Response: Thank you for this comment and to address for this issue, we added the sentence as following:

“This study could provide a deeper understanding of the factors influencing parental decision-making regarding the COVID-19 vaccine, supporting to develop the potential strategies for vaccination plans in the occupational setting.” *(Introduction, Page No. 7, Line No. 152-155)*

Comment 5: You may highlight the novelty of the findings. Does the study reveal new factors influencing hesitancy, or does it align with prior research?

Response: Thanks for these comments. We described the findings for the proportion of attitude towards COVID-19 vaccination and level of parental COVID-19 vaccine hesitancy were the novelty of the findings in this context and comparing the prior research for align and contradict. Moreover, these results and significant associated factors were discussed with the previous studies for whether align or not. *(Discussion, Page No. 24-26, Line No. 467-521)*

The qualitative findings of this study were highlighted and compared with prior studies whether consistent or not, in Discussion section. *(Discussion, Page No. 25, Line No. 492-502)*

Then, we also highlighted the novelty of the findings in the conclusion with the recommendation for the improvement of COVID-19 vaccination plan and increasing the proportion of parental COVID-19 vaccine hesitancy. *(Conclusion, Page No. 28-29, Line No. 572-582)*

3. Methods:

Comment 6: You may clarify how participants were selected for in-depth interviews and whether selection bias may have influenced the qualitative findings.

Response: Thanks for this impressive comment. Sampling method should be consistent with the aims and assumptions in the use of research method. Purposive sampling involves identifying and selecting individuals who were especially knowledgeable about or experienced with a phenomenon of interest. We explored the phenomenon in depth regarding the parental COVID-19 vaccine hesitancy and therefore we used the purposive sampling method for the qualitative method, focusing on the participants who could provide rich information relevant to the research question. To minimize the selection bias, we strived for diverse perspectives in participant section, considering different backgrounds such as age, sex, educational status, and current department.

To get more clarify, we revised as the following:

“To minimize selection bias, participants were selected from diverse backgrounds, including age, sex, educational status, and current department, to capture a wider range of perspectives.” *(Materials and Methods, Page No. 11, Line No. 247-249)*

4. Results:

Comment 7: You may include a comparative table of hesitant versus non-hesitant groups to highlight key differences in demographics and attitudes.

Response: Thank you for this comment. It would have been interesting to explore this aspect. However, we believed that in this study, we could follow our objective by current analysis: identifying the factors related to parental hesitancy to vaccinate their children against COVID-19 (quantitative part) and exploring the reasons for parental COVID-19 vaccine hesitancy among factory workers (qualitative part). The differences of hesitant versus non-hesitant in demographic characteristics and attitudes was expressed in Table 4.

5. Figures:

Comment 8: Figure 1: An in-text citation to is missing. You may mention where it is cited in the text.

Response: Thanks for your comment. We described the in-text citation of Figure 1 (edited as Figure 2) as the following:

“Fig 2 shows the main source of information on the COVID-19 vaccine among the participants: …” *(Results, Page No. 15, Line No. 316-319)*

Comment 9: You may include a flow diagram of participant selection to improve transparency.

Response: Thanks for this suggestion and we agreed with it. The flowchart of participant selection and data collection process is added in the “Participant selection” of manuscript.

“Fig 1 shows the flowchart of participant selection and data collection process of this study.” *(Materials and Methods, Page No. 11, Line No. 249-251)*

Comment 10: You may ensure that figure numbering matches in-text citations.

Response: Thank you for comment and we revised throughout the manuscript.

Minor Comments:

Comment 11: Abbreviations: You may define abbreviations at their first mention (e.g., IDIs at line 231).

Response: Thank you for this comment and we revised it as following:

“In the qualitative approach, individual in-depth-interviews (IDIs) guides were explicitly adapted …” *Materials and Methods, Page No. 11, Line No. 253-254)*

Comment 12: Title: You may consider adding “A Pilot Study” at the end of the title, as the study sample is relatively small.

Response: Thank you for this comment. However, we believe that the current title would be more appropriate because this study aimed to answer specific research questions (to identify the proportion and associated factors of parental COVID-19 vaccine hesitancy and to exploring the reasons to hesitancy among the parents). The pretest and reliability test for the study tools were also performed before the data collection and they were included in Materials and Methods Section. For the small sample size and generalizability, we also mentioned in limitation of the study of Discussion Section.

Comment 13: Tables: You may add a footnote explaining abbreviations and statistical significance levels used in the tables.

Response: Thanks for pointing this out. We agreed with this comment and revised as the foot note in Table 4.

Comment 14: Table 4: You may ensure consistent formatting of the table and fill every cell, if applicable.

Response: Thank you for your suggestion. We would like to explained that in the multivariate analysis columns (AOR and p value columns) of Table 4, the results of multivariate analysis (included two variables in this analysis – sex and infected with the SARS-COV-2) were filled in the cells. We mentioned the analytical procedure in the statistical analysis. Therefore, there were no value in the cells of variables that were not included in this analysis.
