# Supplementary material for: Parental hesitancy on COVID-19 vaccination of children under the age of 16: A cross-sectional mixed-methods study among factory workers
Source: PLoS One. 2025 Jun 26;20(6):e0327056. doi: 10.1371/journal.pone.0327056 (PMC12200862; doi:10.1371/journal.pone.0327056)
Supplement: Attachment — Submitted filename: Response_to_Reviewers_auresp_2.pdf [file pone.0327056.s007.pdf]

<u>Responses to editor and reviewers</u>

PONE-D-25-10068R1

Parental hesitancy on COVID-19 vaccination of children under the age of 16: A cross-sectional mixed-methods study among factory workers

PLOS ONE

Revised manuscript on May 18, 2025

Dear Prof. Harapan Harapan,

I would like to thank you again for the opportunity to revise and resubmit our manuscript titled "Parental hesitancy on COVID-19 vaccination of children under the age of 16: A cross-sectional mixed-methods study among factory workers". We are grateful to you and the reviewers for the helpful comments on our manuscript. It is our belief that the manuscript is substantially improved after making the suggested edits.

Followings this letter are the editor and reviewers' comments with our responses in point by point, including how and where the text was modified. Changes made in the revised manuscript are marked using track changes. The revision has been developed in consultation with all authors, and each author has given approval to the final form of this revision.

Thank you again for consideration of our revised manuscript.

Sincerely,

Ye Minn Htun

Health and Disease Control Unit

Nay Pyi Taw 15011, Myanmar

Journal Requirements

Please review your reference list to ensure that it is complete and correct. If you have cited papers that have been retracted, please include the rationale for doing so in the manuscript text, or remove these references and replace them with relevant current references. Any changes to the reference list should be mentioned in the rebuttal letter that accompanies your revised manuscript. If you need to cite a retracted article, indicate the article's retracted status in the References list and also include a citation and full reference for the retraction notice.

Response: Thanks for this comment. We reviewed and rechecked the references for ensuring that it is correct.

Reviewers' comments:

Review Comments to the Author

Reviewer #1:

Comment: The authours have apprporiately responded to almost all cmoments by the reviewers. However, in line 251 of the revised manuscrpit they refer to Figure 1-Flow chart of participant selection and data collection process. Similarly in line 322 they also refer to Figure 2-main sources of information on COVID--19 Vaccine among participants. The two figures were not included in the manuscript. These had been recommended by one of the reviewers, they should be included.

Response: Thanks for this comment. We prepared and submitted the figures with the separate files, according the submission guideline of PLOS One described as "Do not include figures in the main manuscript file. Each figure must be prepared and submitted as an individual file. Cite figures in ascending numeric order at first appearance in the manuscript file." (https://journals.plos.org/plosone/s/submission-guidelines#loc-figures-and-tables)

(https://journals.plos.org/plosone/s/figures#loc-how-to-submit-figures-and-captions). However, we followed the reviewers' comment and added in the main manuscript (end of the manuscript).

Reviewer #3:

Comment: Thank you for giving me the opportunity to review your manuscript entitled "Parental hesitancy on COVID-19 vaccination of children under the age of 16: A cross-sectional mixed-methods study among factory workers" I very much enjoyed reading your manuscript.

Response: Thanks for your comments on our manuscript.

Comments: Thanks for addressing the previous reviewer comments. I have no further comments.

Response: Thanks for your comments and precious time.
